# Comparative Transcriptome Analysis Reveals Hormone Signal Transduction and Sucrose Metabolism Related Genes Involved in the Regulation of Anther Dehiscence in Photo-Thermo-Sensitive Genic Male Sterile Wheat

**DOI:** 10.3390/biom12081149

**Published:** 2022-08-20

**Authors:** Tianbao Zhang, Shaohua Yuan, Zihan Liu, Liqing Luo, Haoyu Guo, Yanmei Li, Jianfang Bai, Changping Zhao, Liping Zhang

**Affiliations:** Beijing Key Laboratory of Molecular Genetics in Hybrid Wheat, Institute of Hybrid Wheat Beijing Academy of Agriculture and Forestry Science, Beijing 100097, China

**Keywords:** anther dehiscence, hormone, sucrose, male sterility, wheat

## Abstract

Anther dehiscence is an important process to release pollen and then is a critical event in pollination. In the wheat photo-thermo-sensitive genic male sterility (PTGMS) line, pollen cannot release from anther since the anther cannot dehisce during anther dehiscence stage in a sterile condition. In this study, we carried out RNA-sequencing to analyze the transcriptome of one wheat PTGMS line BS366 during anther dehiscence under fertile and sterile conditions to explore the mechanism. We identified 6306 differentially expressed genes (DEGs). Weighted gene co-expression network analysis (WGCNA) and KEGG analysis showed that DEGs were mainly related to “hormone signal transduction pathway” and “starch and sucrose metabolism”. We identified 35 and 23 DEGs related hormone signal transduction and sucrose metabolism, respectively. Compared with conventional wheat Jing411, there were some changes in the contents of hormones, including JA, IAA, BR, ABA and GA3, and sucrose, during three anther dehiscence stages in the sterile condition in BS366. We performed qRT-PCR to verify the expression levels of some critical DEGs of the hormone signaling pathway and the starch and sucrose metabolism pathway. The results showed disparate expression patterns of the critical DEGs of the hormone signaling pathway and the starch and sucrose metabolism pathway in different conditions, suggesting these genes may be involved in the regulation of the anther dehiscence in BS366. Finally, we conducted a hypothesis model to reveal the regulation pathway of hormones and sucrose on anther dehiscence. The information provided new clues to the molecular mechanisms of anther dehiscence in wheat and improved wheat hybrid breeding.

## 1. Introduction

Male sterility has been found in many higher plants, which could apply to the production of hybrid seeds [1]. The lack of functional pollen or the failure of pollen release was associated with male sterility [2]. Pollen is grown within the stamens in flowers. The release of viable pollen from anther is vital for plant reproduction, depending on normal anther dehiscence. Anther dehiscence involves specialized cellular differentiation and degeneration, the anther structural changes, and water status to promote anther opening and pollen release [3]. Anther dehiscence is related to two types of particular cells. One type of specialized cell degrades to make the two lobes of the anther form a single locule. Another type of specialized cell is modified epidermal cells around the stomium, which splits to accelerate anther opening. Anther dehiscence is a process in that the septum breaks down to form a bilocular anther, and then stomium cell breakage and finally pollen release [4].

Many studies showed that anther dehiscence was regulated by the various phytohormones via different pathways. Jasmonic acid (JA) originated from α-linolenic acid, which is derived from phospholipids by the enzyme DEFECTIVE IN ANTHER DEHISCIENCE 1 (DAD1) and next oxygenated by the LYPOXYGENASE (LOX) [5]. OPDA, an oxygenated form, is afterward shifted into JA. The complex of the JASMONATE ZIM DOMAIN (JAZ) and CORONATINE INSENSITIVE 1 (COI1) is a receptor for JA-Ile. The transcription factor MYC2 is suppressed by JAZ proteins. The MYC2 is an activator of jasmonate response genes [6]. It has been reported that JA participates in flower development, pollen vigor, and anther dehiscence [7,8,9,10]. For instance, jasmonates were involved in the maturation of pollens and the dehiscence of the anther, and the defense of wound-induced against biotic attacks in JA biosynthetic mutants [11]. Ectopic expression of *OsJAZ6* influences JA signaling and regulates spikelet development in rice [12]. In wheat, *JAZ*, *COI*, and *MYC* have been reported to be related to anther dehiscence and anther development [9,13,14].

Not only JA, but also other hormones, particularly auxin, were also involved in anther dehiscence and male sterility. During pollen mitosis, downregulation of auxin level was critical for facilitating anther dehiscence together with pollen maturation, especially in the late stamen development [15,16]. The *iaaL* is an indole-3-acetic acid-lysine synthetase gene, which could regulate free auxin content in plants. The *iaaL* transgenic lines presented several abnormalities, including aberrant pollen tubes, the absence of short stamina, the synchronization of stamina development and anther dehiscence, and a diminished seed set [17]. The rice FT-INTERACTING PROTEIN 7 is preferentially expressed in anthers and facilitates nuclear translocation of rice homeobox 1, which inhibits *OsYUCCA4*, a critical auxin biosynthetic gene, in time of the late development stages of anthers [18]. The *arf6 arf8* double mutants could not produce plenty of endogenous JA, and the exogenous JA applications could rescue the anther dehiscence defect [19]. It was found that JA interacts with auxin and could regulate male sterility in the transgenic *Arabidopsis* [20]. The microspore abortion of cytoplasmic male sterile cabbage lines could relate to the excessive ABA content in the anthers and leaves [21]. Finally, exogenous GA-treated wild-type *Arabidopsis* lines and double mutants in GAI and RGA, two repressors of the GA signaling, showed a loss of fertility [22].

Sugar, as an essential energy source, has been shown to play a significant role in plant male reproduction. During anther development, flaws in sugar metabolism could make for male sterility [23,24]. In the presence of sugars derived from starch, mature pollen could germinate smoothly and facilitate pollen tube cell wall formation [25]. Therefore, starch and sugar metabolism are needed for anther dehiscence pollen germination, and pollen tube growth [24,26]. For instance, disruption of rice *SUCROSE PHOSPHATE SYNTHASE 1* (*OsSPS1*), coding a rate-limiting enzyme in the process of sucrose synthesis, exhibited abnormal pollen germination and resulted in male sterility in *Oryza sativa* [27]. RNAi-mediated inhibition of *OsHXK10*, a hexokinase gene, resulted in anther indehiscence and a reduction in the pollen germination [28]. Pectinases, cellulases, and hemicellulases in plants, for example, β-1,4-glycosidases, PGs, and extensin could regulate organ dehiscence and abscission, since they could involve in anther cell walls modification and degradation [29]. Cellulose, a component part of the pollen wall, is required for microspores development and maturation [30]. The glycoside hydrolase family genes, such as Glycoside hydrolase family 9 (GH9), are required for pollen wall development during late anther development, containing anther dehiscence [31]. GH9 is a critical member of the hydrolase family regulating cellulose synthesis and hydrolysis, playing an essential role in pollen fertility conversion and anther dehiscence [32]. Therefore, more male sterility genes related to sugar metabolism require to be identified deeply in diverse plants, especially wheat.

Wheat PTGMS lines are critical female parents in a two-line hybrid breeding system. The fertility of a wheat PTGMS line BS366 is affected by photoperiod and temperature. When the photoperiod is long-day (longer than 14 h:10 h light:dark photoperiod), or the average environment temperature is lower than 20 °C during the pollen mother cell stage to the meiosis stage, the wheat PTGMS line BS366 exhibit male fertility. When the photoperiod is short-day (shorter than 10 h:14 h light:dark photoperiod), or the average environment temperature is lower than 12 °C during the pollen mother cell stage to the meiosis stage, the wheat PTGMS line BS366 exhibit male sterility. Previous studies have shown that male sterility in the wheat PTGMS line BS366 is mainly due to pollen sterility and anther indehiscence [33,34]. Morphological evidence had indicated that pollen cells of BS366 were sterile, and anthers lost the ability to dehisce under a sterile environment [14,35]. Therefore, to discover the genes from the wheat that may be involved in the control of anther dehiscence and to reveal the regulatory pathway of anther dehiscence, RNA-sequencing was used for anthers during anther dehiscence stages. WGCNA analysis was performed to isolate the critical gene module regulating anther dehiscence. A sufficient understanding of anther dehiscence will extend the content of mechanisms for regulating male fertility.

## 2. Results

### 2.1. Phenotypic Characterization of Anther Dehiscence

**Figure 1:**
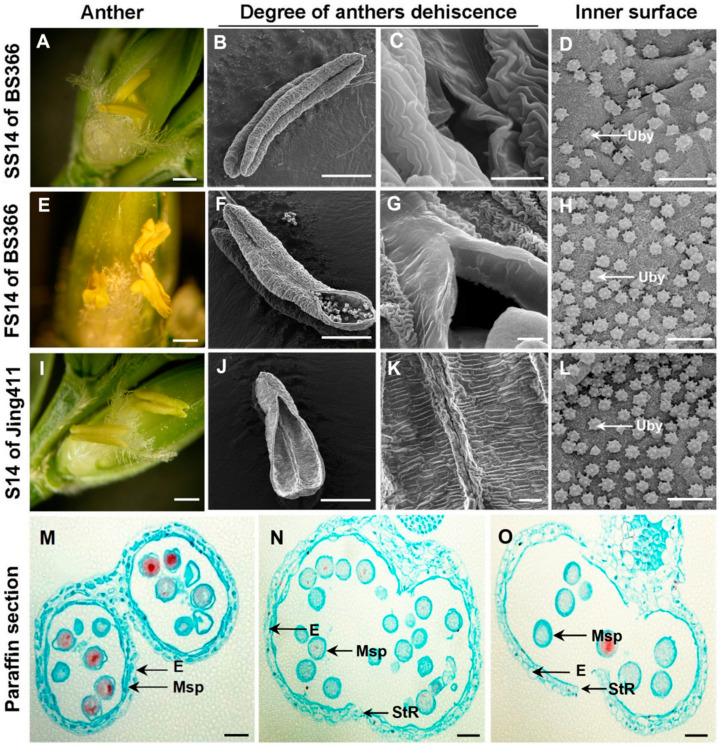
Morphological characterization of anther from BS366 of different fertility conditions and Jing411. (**A**) Anthers of BS366 at the dehiscence stage from the sterile condition were photographed with a dissecting microscope. (**B**–**D**) Anthers and the inner surface of BS366 at the dehiscence stage from the sterile condition were photographed with by scanning electron microscope. (**E**) Anthers of BS366 at the dehiscence stage from the fertile condition were photographed with a dissecting microscope. (**F**–**H**) Anthers and the inner surface of BS366 at the dehiscence stage from the fertile condition were photographed with a scanning electron microscope. (**I**) Anthers of Jing411 at the dehiscence stage were photographed with a dissecting microscope. (**J**–**L**) Anthers and inner surface of Jing411 and at the dehiscence stage were photographed with a scanning electron microscope. (**M**–**O**) Cytological observation on the internal structure of anthers from sterile BS366 (**M**), fertile BS366 (**N**), and Jing411 (**O**), respectively. E, epidermis; Msp, microspores; StR, stomium region; Uby, Ubisch bodies. Scale bars represent 1 mm in anthers (**A**,**B**,**E**,**F**,**I**,**J**), 10 μm on (**C**,**D**,**G**,**H**,**K**,**L**), and 50 μm on (**M**–**O**).

Male sterility is an effective means for the use of wheat heterosis. Anther indehiscence is one of the main forms of male sterility. The results of morphological observation showed that in stage 14 (dehiscence stage anther) of anther development of BS366 under fertile conditions (FS14), the anthers produced small stomiums to release pollens on the day of flowering (Figure 1E–G), while the degree of anther dehiscence in FS14 of BS366 was smaller than the anthers in stage 14 of common wheat Jing411 (Figure 1I–K). In contrast, the anthers in stage 14 of anther development of BS366 under sterile conditions (SS14) were indehiscence and without pollen releasing (Figure 1A–C). Moreover, compared with BS366 of fertile condition and Jing411, the ubisch bodies on the inner epidermis of BS366 of sterile condition were accumulated fewer and more sparsely distributed (Figure 1D,H,L). To further observe the internal structure of anther cytology, we used paraffin sections to find the anther septum of Jing411 degraded, and the cells in the stomium region were completely dehiscent at the crucial stage of anther dehiscence (stage 14) (Figure 1O). Moreover, anthers of BS366 under fertile conditions showed a crack, and the pollen grains mainly released from the crack could be observed (Figure 1N). However, the anther wall of BS366, under sterile conditions, collapsed inward, containing a large number of pollen grains and no obvious dehiscence (Figure 1M). In addition, there were underdeveloped grains of irregular shape, and pollen with a large nucleus located in the center, which might indicate a violation of microspore division (Figure 1M).

### 2.2. Overview of RNA-Seq Data Analysis

**Figure 2:**
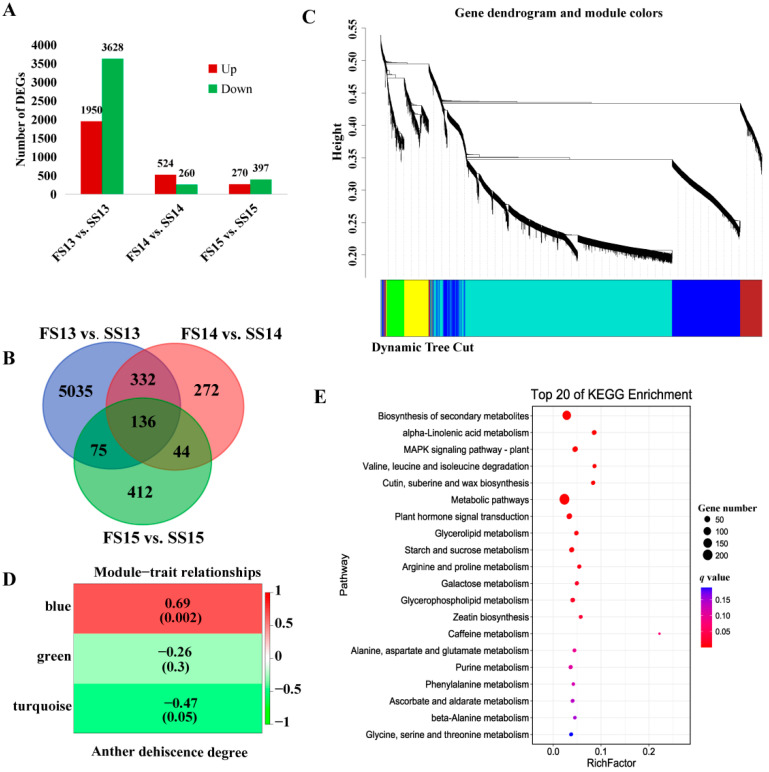
Identification of differentially expressed genes and weighted gene co-expression network analysis of DEGs. (**A**) Analysis of DEGs between fertile and sterile conditions in BS366. (**B**) Venn of different stages of DEGs between fertile and sterile conditions. (**C**) Genes of dendrogram and module colors of the WGCNA. (**D**) Module–trait relationships of the WGCNA. The blue module, green module, and turquoise module represent three modules from Figure 2C. The colors of panels show a color scale for module–trait relationships ranging from −1 to 1. (**E**) The top20 of KEGG enrichment of the DEGs of the blue module of WGCNA.

To investigate the molecular mechanism in abnormal anther dehiscence of BS366 of different fertility conditions, transcriptomics analysis was performed by RNA-Seq using anther samples in stages 13, 14, and 15 under fertile (named FS13, FS14, and FS15 for each developmental stage, respectively), and sterile conditions (named SS13, SS14, and SS15 for each developmental stage, respectively) (Figure 2A). As a result, a mean of 10.28 Gb clean reads from each sample was screened following a series of data filtering. Among 18 samples, the average Q20 value was higher than 96.2%. The proportion of the GC accounted for 52.56% (Table 1). There were 85,280 genes expressed in the samples (Table S1).

### 2.3. Identification of Differentially Expressed Genes and Weighted Gene Co-Expression Network Analysis for DEGs

The expression values of genes in samples were normalized by the fragments per kilobase of exon model per million reads mapped (FPKM) algorithm and statistically analyzed. After computing the expression values, 5578, 784 and 667 DEGs exhibited different expressions between FS13 vs. SS13, FS14 vs. SS14, and FS15 vs. SS15 libraries, respectively. There were 5578 DEGs detected between FS13 vs. SS13 libraries, of which 3628 were downregulated and 1950 were upregulated. A total of 784 DEGs in FS14 vs. SS14 libraries, among which 260 were downregulated and 524 were upregulated. For 667 DEGs identified in FS15 vs. SS15 libraries, 397 were downregulated and 270 were upregulated (Figure 2A).

In order to identify the candidate DEGs involved in regulating anther dehiscence, we analyzed the DEGs in different libraries. Among all the 6306 identified DEGs, 136 genes co-expressed between FS13 vs. SS13, FS14 vs. SS14, and FS15 vs. SS15 libraries of anther development. A total of 5035 genes, were specifically expressed between FS13 vs. SS13 library and 272 genes specifically expressed between the FS14 vs. SS14 library, while there were 412 genes specifically expressed between the FS15 vs. SS15 library (Figure 2B).

WGCNA was performed using all of the 6306 DEGs to further elucidate the genes associated with anther dehiscence. Analysis of the module–trait relationships using the anther dehiscence degree as the trait data showed that the “blue” module (r = 0.69, *p* = 0.002) was highly positive correlation with anther dehiscence (Figure 2C,D). In order to understand the functions of the key DEGs of the blue module, a KEGG pathway analysis was carried out (Figure 2E). Significant pathway enrichment analysis exhibited that plant hormone signal transduction pathway and starch and sucrose metabolism pathway were the pivotal biological events in the process of anther dehiscence. 

### 2.4. Expression Patterns of DEGs of the Plant Hormone Signal Transduction Pathway and Endogenous Hormone Measurements

**Figure 3:**
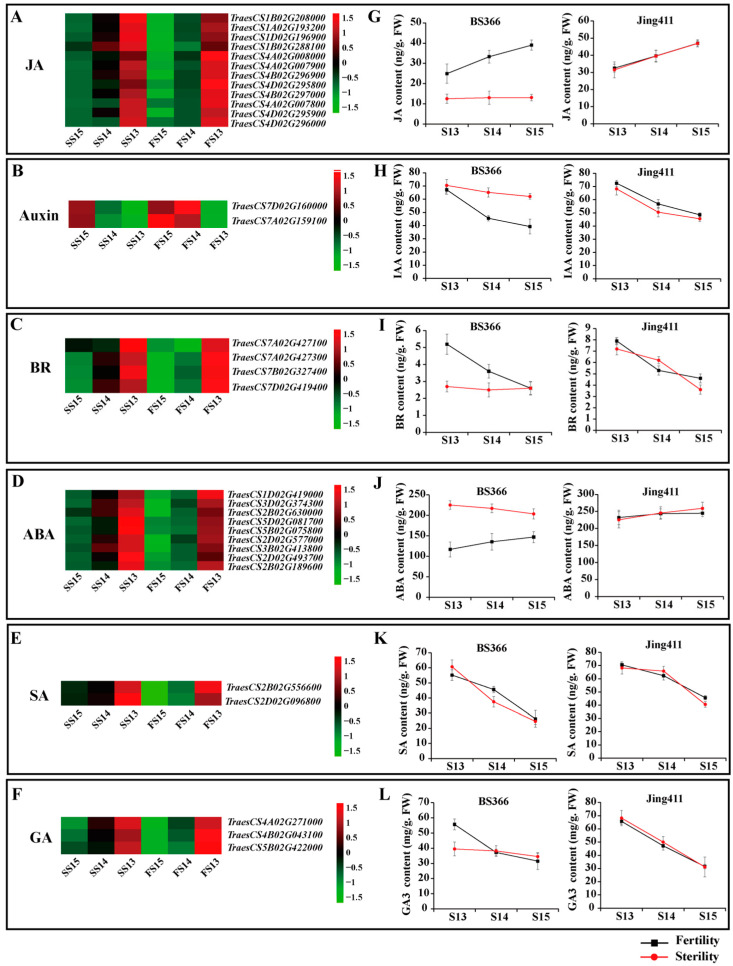
Expression patterns of plant hormone-related DEGs in the blue module of WGCNA and hormone contents at anther dehiscence stage in BS366 and Jing411 under fertile and sterile conditions. (**A**–**F**) are heatmaps for JA, Auxin (IAA), BR, ABA, SA, and GA (GA3), respectively. (**G**–**L**) Are contents of JA, Auxin (IAA), BR, ABA, SA, and GA (GA3), respectively.

In order to understand the hormonal regulation of abnormal anther dehiscence in more detail, the expression levels of pivotal DEGs in the jasmonate acid, auxin, brassinosteroid, abscisic acid, salicylic acid, and gibberellin signaling pathways were analyzed. During later anther development, the expression levels of most DEGs in the jasmonate acid signaling pathways were gradually rising. In stage 14, most DEGs in the jasmonate acid signaling pathways were highly expressed under fertile conditions than sterile conditions. During anther dehiscence, DEGs in the auxin signaling pathway were downregulated expression. The expression levels of two DEGs in the auxin signaling pathway under fertile conditions were lower than that under sterile conditions. Four genes in the BR signaling pathway were differentially expressed. Nine DEGs in the ABA signaling pathway were identified. Two genes in the SA signaling pathway were DEGs. Three DEGs in the GA signaling pathway were upregulated in expression during anther development. The three DEGs in the GA signaling pathway exhibited higher expression levels under fertile conditions (Figure 3).

We measured the contents of endogenous hormones, including JA, IAA, BR, ABA, SA, and GA3 of BS366 and Jing411 under fertile and sterile conditions during the late stage of anther development. In BS366, JA levels under sterile conditions changed little and were lower than JA levels under fertile conditions, while the levels of JA under fertile conditions were gradually increasing as the anther developed. In Jing411, the levels of JA under two conditions were increasing, and no difference. The IAA contents were gradually reduced during stages 13 to 15 of anther development of BS366 under fertile conditions, which was consistent with the dynamic change of IAA in Jing411 under fertile and sterile conditions. However, the levels of IAA in BS366 from stage 14 to 15 of anther development under sterile conditions were higher than that under fertile conditions. There was a gradual decrease in BR contents of Jing411 under two conditions. However, the BR contents of BS366 under fertile conditions were reduced, while those under sterile conditions were no significant change. The ABA contents in BS366 from stages 13 to 15 under sterile conditions were higher than those under fertile conditions, while there was no difference in ABA content between the two conditions in Jing411. The levels of SA in Jing411 were gradually decreasing, but no significant difference between the two conditions. The SA contents under two conditions were also decreased during anther development, but at stage 14 of BS366, the SA contents under fertile conditions were a little higher than that under sterile conditions. The levels of GA3 in Jing411 reduced from stage 13 to 15 of anther development. However, the levels of GA3 in BS366 did not change much during anther development under sterile conditions, while the levels of GA3 under fertile conditions had a downward trend (Figure 3).

### 2.5. Expression Patterns of DEGs of Starch and Sucrose Metabolism Pathway and the Sucrose Content of Anthers Measurements

**Figure 4:**
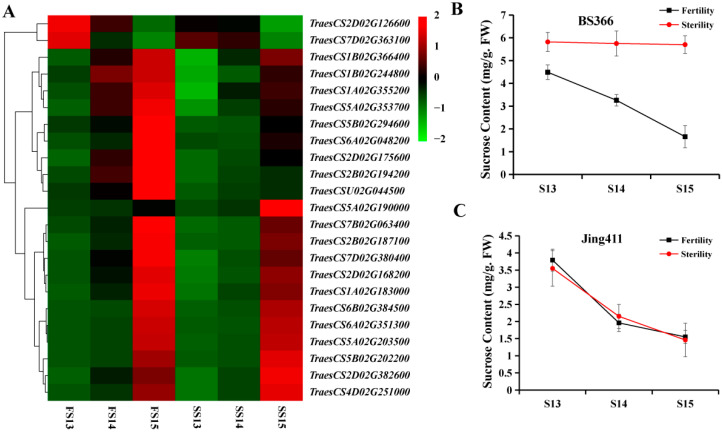
Expression levels of DEGs of the starch and sucrose metabolism pathway of BS366 in the blue module of WGCNA and sucrose contents of BS366 and Jing411 under fertile and sterile conditions. (**A**) Heatmap of the DEGs of the starch and sucrose metabolism pathway of BS366 in the blue module of WGCNA. (**B**) Sucrose contents of anthers of BS366 under fertile and sterile conditions during the later anther development. (**C**) Sucrose contents of anthers of Jing411 under fertile and sterile conditions during the later anther development.

To investigate the starch and sucrose metabolism pathway controlling anther indehiscence in greater detail, the expression levels of key DEGs in the starch and sucrose metabolism pathways were analyzed. Twenty-three genes in the starch and sucrose metabolism pathway were differentially expressed between fertile and sterile conditions. Most of the DEGs in starch and sucrose metabolism were upregulated during the development of anther (Figure 4A).

The sucrose contents of anthers were determined during the period of flowering (Figure 4B,C). The results showed that the sucrose contents of anthers of Jing411 were identical under two conditions. However, the sucrose contents of anthers of BS366 were declining during the period of flowering under fertile conditions, while sucrose contents were no longer declining under sterile conditions, suggesting that sucrose has a significant effect on anther dehiscence. The results were highly consistent with the trend of expression of *TraesCSU02G044500*, *TraesCS2B02G194200*, and *TraesCS2D02G175600* genes during the anther dehiscence stages. 

### 2.6. Validation of DEGs by qRT-PCR

**Figure 5:**
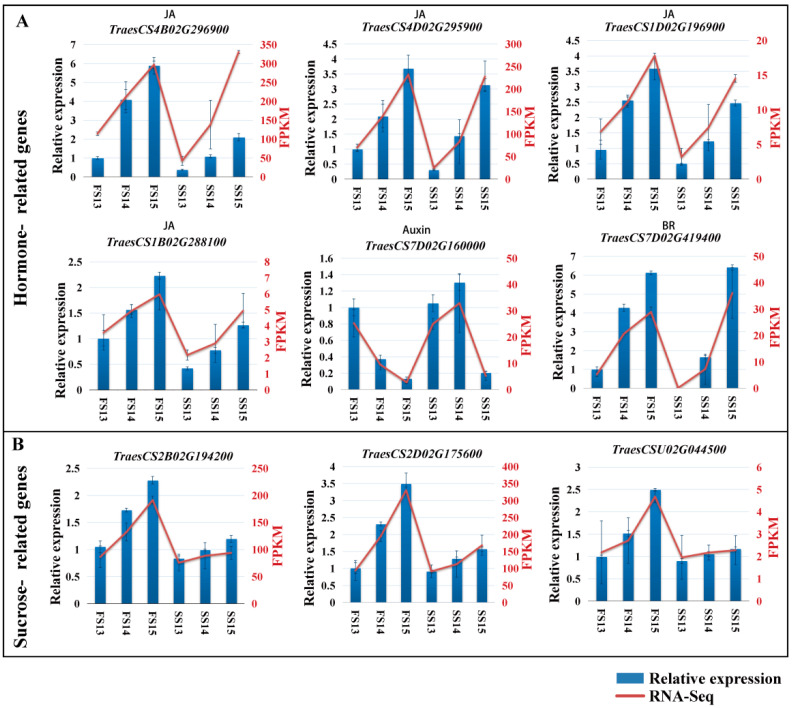
qRT-PCR analysis of the expression patterns of randomly selected hormone- (**A**) and sucrose (**B**)-related DEGs.

Anther samples were collected from the fertile and sterile plant and the expression levels of some key genes of the hormone signaling pathway and the starch and sucrose metabolism pathway were measured by qRT-PCR. The expression of six genes of the hormone signaling pathway and three genes of the starch and sucrose metabolism were analyzed by qRT-PCR (Figure 5). The expression tendencies were consistent with the RNA-Seq results, indicating that the transcriptome sequencing results were accurate and reliable in this study. 

## 3. Discussion

Anther dehiscence contains multiple processes and exists in the late stages of anther development, which affects sexual reproduction through the well-timed release of mature pollen grains for fertilization of flowering plants (Song, 2018). Abnormal anther dehiscence is a key factor for male sterility. To make clear the types and amounts of DEGs with reference to controlling anther dehiscence process of wheat, the important and common DEGs were discovered. The results of this study revealed that DEGs involved in the hormone signal transduction pathway and the starch and sucrose metabolism were identified by transcriptome between the anther-indehiscent wheat and the anther-dehiscent wheat.

Plant development and responses to multiple environmental signals are regulated by sophisticated multicomponent signaling networks. JA is a key component of the regulatory system. JA takes part in most stages of growth and development of plants and also controls anther dehiscence [36,37]. Studies on the JA signaling pathway also reveal the diversified functions of JA during plant reproduction. Plants separately overexpressing *mJAZ3*, *mJAZ4*, *mJAZ6*, *mJAZ7*, and *mJAZ11*, respectively, containing amino acid substitutions in the JAZ domain, showed low fertility and defective development of spikelet [38]. *OsCOI1b* could regulate spikelet development by interacting with *OsJAZ1/EG2* [8]. These investigations indicate a common JA signaling pathway with multiple roles in rice and *Arabidopsis* in reproductive development. In our study, we found some JAZs were differentially expressed in the anther dehiscence process, such as *TraesCS4B02G296900* and *TraesCS4B02G297000*. The transcriptome data showed that the feedback control of JA signaling in abnormal anther dehiscent wheat altered the expression patterns of genes at the mRNA level during anther development. 

Auxin participates in the late stamen developmental stages, negatively controls pollen maturation and anther dehiscence, and facilitates filament elongation [16]. Auxin-responsive reporter genes are expressed in anthers and can be induced by an exogenous auxin [15]. In the study, genes of the auxin signaling pathway were differentially expressed between fertile and sterile conditions. Marciniak’s results revealed that GA3 contributed to the control of anther dehiscence process in yellow lupine [39]. In our study, some genes of the GA signaling pathway were differentially expressed during anther dehiscence period under two conditions. In addition, our results exhibited that genes of BR, ABA, and SA signaling pathways might also involve anther dehiscence.

Chloroplasts in endothecium cells of anther showed active photosynthetic ability in order to enable starch metabolism and carbohydrate supply in the later stages of anther development in maize [40]. Some flowers’ anthers in the RNAi lines were non-dehiscent by suppressing *OsHXK10* (Hexokinase) expression in rice [41]. Sucrose is transported via the terminal phloem to the outer wall layers of anther and transferred across the middle layer by way of varieties of sugar transporters to anther tapetum [24]. Experimental data in this study showed that genes of sucrose synthase (*TraesCS2B02G194200* and *TraesCS2D02G175600*) and sucrose phosphate synthase (*TraesCSU02G044500*) had different expressions between anther-dehiscent wheat and the anther-indehiscent wheat. Sucrose contents were also consistent with the expression levels of DEGs. These results reveal that sucrose may play a significant role in anther dehiscence in PTGMS wheat. 

Aquaporins, members of membrane intrinsic proteins, can efficiently transport water and several small molecules. Plasma membrane intrinsic proteins (PIPs) involve in regulating water absorption and efflux in plant cells. Beta glucosidase (BG), a kind of hydrolase, participates in hydrolyzing glycosidic bonds from oligosaccharides or glycopolymers so as to release the non-reducing sugar group, which plays a critical role in the regulation of anther dehiscence. The expression of *TraesCS7B02G289100* in the sterile anther was higher than that in the fertile environment. Higher expression of *TraesCS7B02G289100* could enhance the soluble sugar content within the anther under the sterile condition, and then raise the osmotic potential of anthers, consequently slowing down the dehydration of anther dehiscence. 

### Putative Anther Dehiscence Related Male Sterile Network in Wheat PTGMS Line

**Figure 6:**
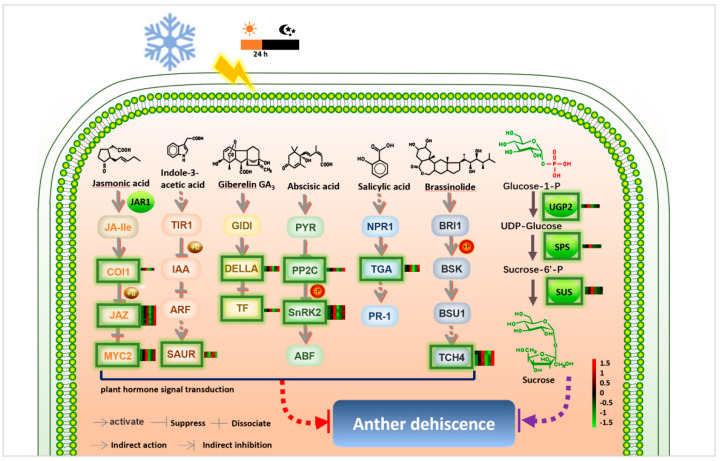
A hypothetical model of hormones and sucrose regulating anther dehiscence in wheat. Abbreviations: JAR1, jasmonic acid-amino synthetase; COI1, coronatine-insensitive protein 1; JAZ, jasmonate ZIM domain-containing protein; MYC2, transcription factor MYC2; TIR1, transport inhibitor response 1; IAA, auxin-responsive protein IAA; ARF, auxin response factor; SAUR, SAUR family protein; GIDI, gibberellin receptor GID1; DELLA, DELLA protein; TF(PIF4), phytochrome-interacting factor 4; PYR, abscisic acid receptor PYR/PYL family; PP2C, protein phosphatase 2C; SnRK2, serine/threonine-protein kinase SRK2; ABF, ABA-responsive element binding factor; NPR1, regulatory protein NPR1; TGA, transcription factor TGA; PR1, pathogenesis-related protein 1; BRI1, protein brassinosteroid insensitive 1; BSK, BR-signaling kinase; BSU1, serine/threonine-protein phosphatase BSU1; TCH4, xyloglucan:xyloglucosyl transferase TCH4; UGP2, UTP—glucose-1-phosphate uridylyltransferase; SPS, sucrose-phosphate synthase; SUS, sucrose synthase. +u, ubiquitylation; +p, phosphorylation; −p, dephosphorylation.

According to the above results and previously published results, we proposed a putative model of anther dehiscence regulated by hormones and sucrose in wheat (Figure 6). If experiencing low temperature and short-day conditions during the meiosis stage (fertility-sensitive stage), the anthers of the BS366 would show abnormal dehiscence. The hormone was one of the critical effectors that regulated the anther dehiscence [42]. JA is the fastest signal molecule for plants to respond to external stimuli in the growth and development stages of plants and defense responses to biological and abiotic stresses [43]. As shown in Figure 6, low levels of JA may lead to decreased expression of genes encoding coronatine-insensitive protein 1, jasmonate ZIM domain-containing protein, and basic helix-loop-helix (bHLH) DNA-binding family protein (*COI1*, *JAZ*, and *MYC2*), then affected the anther dehiscence. Furthermore, the increase in auxin contents at stages 14 and 15 may regulate the expression level of *SAUR* (encoding SAUR family protein) and then may affect anther dehiscence. The expression levels of the genes encoding DELLA protein and phytochrome-interacting factor 4 were downregulated due to the change of GA contents may cause abnormal anther dehiscence. ABA, as a “stress hormone”, not only participates in the regulation of various stages of plant growth and development but also plays a key role in the process of plants responding to various environmental stresses. The double negative regulatory system in the ABA signal transduction pathway -*PYR*/*PYL*-|*PP2C*-|*SnRK2*-*ABF* regulates ABA signal transduction and its downstream response [44]. The abnormal accumulation of ABA under low temperature and short-day stimulation may lead to the downregulation of genes encoding serine/threonine-protein kinase (*SnRK2*) and protein phosphatase 2C (*PP2C*) and then may affect the anther dehiscence. Under the influence of the environment, the decrease in BR contents in stages 13 and 14 may lead to a decrease in the expression level of *TCH4* encoding xyloglucosyl transferase. Sucrose was another important effector controlling anther dehiscence. UTP-glucose-1-phosphate uridylyltransferase participates in the synthesis of plant sucrose and sugar metabolism, which is essential for anther cell wall formation [45]. The expression levels of *UGP2*, *SPS*, and *SUS* genes were changed, and then sucrose content was regulated. The change of sucrose content may result in the change of osmotic potential in anther cell, which causes the anther indehiscence. Therefore, the complexity of plant hormones and sucrose metabolism-related pathways provides an excellent buffer system for plants to control the sophisticated process of anther dehiscence.

## 4. Materials and Methods

### 4.1. Plant Material

Wheat photo-thermo-sensitive male sterile (PTGMS) line BS366 and conventional wheat Jing411 were used in the study. BS366, a winter wheat, was a doubled haploid line (progeny of Jingnong8121/E8075-7) [32]. Jing411 was a conventional wheat (progeny of Fengkang2/Changfeng5). The wheat was grown in Dengzhou (China, 32°67′ N, 112°16′ E) and Beijing (China, 39°54′ N, 116°18′ E) under natural conditions, and then managed routinely. The anther samples from Beijing (fertile condition) and Dengzhou (sterile condition), were collected at different developmental stages (S13: Bilocular stage anther, S14: Dehiscence stage anther, S15: Senescence stage anther) [46] and named FS13, FS14, FS15, SS13, SS14 and SS15 for each developmental stage, respectively.

### 4.2. Phenotypic Characterization at the Dehiscence Stage

Anthers at the dehiscence stage from Dengzhou and Beijing conditions were photographed with a dissecting microscope (ZEISS SteREO Discovery.V20; ZEISS, Jena, Germany) and scanned with a scanning electron microscope (SEM) (Phenom LE, Wetzlar, Germany) after treating with 2.5% glutaraldehyde, according to the reported method [47]. 

### 4.3. Measurement of Plant Endogenous Hormones

The contents of endogenous hormones, including JA, Auxin, GA, SA, BR, and ABA, were measured by the ELISA method with related kits according to the manufacturer’s instructions (Comin, Jiangsu, China). Each sample was repeatedly measured three times.

### 4.4. Measure the Concentration of Sucrose

A total of 1000 mg of fresh wheat anther tissues was frozen in liquid nitrogen and well ground using the organization grinding apparatus in a 2 mL centrifuge tube. Following the addition of 5 mL 80% ethanol, tissue homogenates were heated in an 80 °C water bath for 30 min while vibrating constantly. After being cooled, the compound was centrifuged at 3500× *g* for 10 min, and then the supernate was transferred to 25 mL volumetric flask. Adding 5 mL 80% ethanol to elute precipitate two times, and then gathered eluent into volumetric flask, diluted with 80% ethanol solution to volume, and mixed [48].

### 4.5. RNA Extraction and Sequencing

Total RNA was extracted utilizing TRIzol reagent (Invitrogen, Nottingham, UK) following the manufacturer’s instructions. The concentrations of purified RNA were determined using a NanoDrop UV–visible spectrophotometer (Thermo Fisher, Waltham, MA, USA). Transcriptome library conduction was performed as previously described [49]. Sequencing was accomplished using the Illumina HiSeq2500 sequencing platform (Igenecode, Beijing, China). All the transcriptome raw data have been deposited in National Genomics Data Center (Beijing, China), Genome Sequence Archive (GSA) database with the Accession number: (CRA006881).

### 4.6. Bioinformatic Analysis of Transcriptome Data

RNA library sequencing transcript assembly was performed by Igenecode Ltd. based on the HISAT method [50]. Assembled reads were mapped to the published genome of Chinese Spring (https://plants.ensembl.org/Triticum_aestivum/Info/Index?db=core, accessed on 3 June 2019). Fragments per kilobase of exon model per million mapped fragments (FPKM) were calculated to describe the gene expression levels. The DEGs were identified by applying a cutoff log2 (fold change) of >1 and <−1 for upregulated and downregulated genes, respectively, with *p* < 0.05, following the previous study [51]. The WGCNA was performed for the DEGs by using the R package of WGCNA to infer the gene co-expression modules that had a strong correlation with anther dehiscence [52]. The adjacency matrix was obtained based on the pairwise Pearson’s correlation coefficients between pairs of genes. Construction of the WGCNA network and module testing was performed using an unsigned type of topology overlap matrix, with a power β of 23 and a branch merge cut height of 0.25. The module eigengene (the first principal component of a given module) was calculated and used to evaluate the module’s correlation with anther dehiscence. KEGG (Kyoto Encyclopedia of Genes and Genomes) pathway analysis was performed to reveal the gene function in the blue module in this study [53]. KEGG enrichment analysis with a corrected *p*-value ≤ 0.05 were considered to be significantly enriched [49].

### 4.7. qRT-PCR Analysis

The relative expression levels of selected genes were determined using TB Green^®^ Premix Ex Taq™ (TaKaRa, Shiga, Japan) after cDNA was synthesized [32]. The qRT-PCR reaction was performed on a 10 µL scale using SYBR Premix Ex Taq™ (TaKaRa, Shiga, Japan) on an Eco Real-Time PCR System (Illumina, CA, USA). The relative expression levels were calculated by the 2^−∆∆Ct^ method [54]. Each sample had three independent biological replicates, and each biological replicate comprised three technical replicates. The *Actin* gene (GenBank accession: 542814) in wheat was used as a reference gene. The primers used in the study were designed by the Primer premier 5.0 (Primer, CA, USA) program and then listed in Table S2.

### 4.8. A putative Model of Anther Dehiscence in Wheat PTGMS Line

We conducted a hypothesis model of anther dehiscence in wheat PTGMS line based on the KEGG pathway (ID 04075 and 00500), the expression level of hormone- and sucrose-related DEGs, and the contents of the hormones and sucrose, and previous studies. The related DEGs contain *COI1*, *JAZ*, *MYC2*, *SAUR*, *DELLA*, *TF*, *PP2C*, *SnRK2*, *TGA*, *TCH4*, *UGP2*, *SPS* and *SUS* [9,13,14,42,43,44,45]. The expression levels of the above-mentioned DEGs were shown beside them in the figure. We used the software of Adobe Illustrator CS5 (Adobe, CA, USA) to draw this figure.

## 5. Conclusions

In this study, we analyzed the transcriptomes of one wheat PTGMS line BS366 during anther dehiscence under fertile and sterile conditions to explore the molecular mechanism of anther dehiscence. We discovered the genes in the hormone signal transduction pathway and starch and sucrose metabolism from the wheat that may be involved in the control of anther dehiscence. Thirty-five DEGs related to hormone signal transduction and twenty-three DEGs related to sucrose metabolism may be associated with anther dehiscence. These genes provide a rich resource for future studies on anther dehiscence. Additionally, the contents of JA, Auxin, BR, ABA, SA, GA and sucrose of BS366 were different in two environments, suggesting that these hormones and sucrose might play a key role in regulating anther dehiscence. These results shed new light on revealing the molecular regulatory mechanism of anther dehiscence and male sterility.

## Figures and Tables

**Table 1:** Statistical results of RNA sequencing data.

Sample	Clean Reads (M)	Clean Bases (G)	Q20 (%)	GC (%)	Total Mapped	Unique Mapped
FS13-1	70.1261M	10.5189G	96.57	52.07	67,399,956 (96.11%)	62,945,694 (89.76%)
FS13-2	69.2820M	10.3923G	96.21	52.66	66,795,338 (96.41%)	62,356,024 (90.00%)
FS13-3	68.0320M	10.2048G	96.59	52.42	65,494,818 (96.27%)	61,426,150 (90.29%)
FS14-1	67.8116M	10.1717G	96.12	53.43	65,422,558 (96.48%)	60,944,936 (89.87%)
FS14-2	67.4691M	10.1204G	96.37	52.56	65,022,032 (96.37%)	60,901,854 (90.27%)
FS14-3	69.3257M	10.3989G	96	52.99	66,711,060 (96.23%)	62,484,910 (90.13%)
FS15-1	67.7965M	10.1695G	95.72	53.46	65,353,484 (96.40%)	61,362,940 (90.51%)
FS15-2	69.3829M	10.4074G	96.1	53.35	66,889,454 (96.41%)	62,665,108 (90.32%)
FS15-3	71.1352M	10.6703G	96.37	53.42	68,578,804 (96.41%)	64,320,258 (90.42%)
SS13-1	69.1204M	10.3681G	96.46	51.5	66,420,770 (96.09%)	62,481,716 (90.40%)
SS13-2	69.3102M	10.3965G	96.92	50.86	66,429,536 (95.84%)	62,370,380 (89.99%)
SS13-3	67.8598M	10.1790G	96.96	51.49	65,341,338 (96.29%)	61,350,318 (90.41%)
SS14-1	71.0547M	10.6582G	96.46	51.73	68,495,044 (96.40%)	64,330,860 (90.54%)
SS14-2	68.4178M	10.2627G	95.8	52.68	65,398,718 (95.59%)	61,288,724 (89.58%)
SS14-3	63.8187M	9.5728G	95.87	52.49	61,324,110 (96.09%)	57,427,232 (89.99%)
SS15-1	64.4363M	9.6654G	95.69	52.87	61,858,806 (96.00%)	57,772,684 (89.66%)
SS15-2	69.6364M	10.4455G	95.65	53.26	66,919,980 (96.10%)	62,606,924 (89.91%)
SS15-3	69.5348M	10.4302G	95.86	52.76	66,832,700 (96.11%)	62,456,070 (89.82%)

## Data Availability

Not applicable.

## Supplementary Materials

The following supporting information can be downloaded at: https://www.mdpi.com/article/10.3390/biom12081149/s1. Table S1: The expression of gene of the 18 libraries; Table S2: qRT-PCR primer sequences.

## Abbreviations

ABA	Abscisic acid
BR	Brassinolide
DEGs	Differentially expressed genes
FPKM	Fragments per kilobase of exon model per million reads mapped
GA	Gibberellin acid
IAA	Indoleacetic acid
JA	Jasmonic acid
KEGG	Kyoto Encyclopedia of Genes and Genomes
PTGMS	Photo-thermo-sensitive genic male sterility
qRT-PCR	Quantitative real-time polymerase chain reaction
SEM	Scanning electron microscope
SA	Salicylic acid
WGCNA	Weighted gene co-expression network analysis
