# Supplementary material for: Comparative Transcriptome Analysis Reveals Hormone Signal Transduction and Sucrose Metabolism Related Genes Involved in the Regulation of Anther Dehiscence in Photo-Thermo-Sensitive Genic Male Sterile Wheat
Source: Biomolecules. 2022 Aug 20;12(8):1149. doi: 10.3390/biom12081149 (PMC9406143; doi:10.3390/biom12081149)
Supplement: Supplementary file 1 [file biomolecules-12-01149-s001.zip › Table S2 qRT-PCR primer sequences.pdf]

Table S2 qRT-PCR primer sequences

Genes	Primers (5' → 3')
Actin	F: CAGGCAACCAACGCCTTTAC
	R: AAATCTGTTCACGGGAGCGT
TraesCS4B02G296900	F: GCTGACGATCTTCTACGGGG
	R: CTTGCGCTTCGACAGGAAC
TraesCS4D02G295900	F: CAGTACATGAGGGAGCAGCC
	R: GGGAAGTCGTCGAACACGAA
TraesCS1D02G196900	F: GCTCACGGAGAACCCCAG
	R: CTTGAAGAAAGGCGGGGTCA
TraesCS7D02G160000	F: GGGAGTAATGCTCACGGGAC
	R: TGTGCTCCTCCTCCTTCTCC
TraesCS1B02G288100	F: GGAGCTCAAGCTCGACAAGT
	R: ACTTGCAATTCCTGGCGAGA
TraesCS7D02G419400	F: GAGACGTCCTCACGCTGTC
	R: ACAGGTAGAAAGTGGCGACG
TraesCS2B02G194200	F: TGCTTCCCAAAGCACAACCT
	R: GACAAGCTCATTGGTGTGCG
TraesCS2D02G175600	F: CAATTCTCCCTCGAGTCCCTG
	R: GGATCGCGGCATTGTACTCA
TraesCSU02G044500	F: TGATTCAGATACAGGCGGTC
	R: GGCCCAAATGGTATCCTGAC
